# Interface-induced spontaneous positive and conventional negative exchange bias effects in bilayer La_0.7_Sr_0.3_MnO_3_/Eu_0.45_Sr_0.55_MnO_3_ heterostructures

**DOI:** 10.1038/s41598-017-07033-x

**Published:** 2017-07-31

**Authors:** J. Krishna Murthy, P. S. Anil Kumar

**Affiliations:** Department of Physics, Indian Institute of Science, Bengaluru, 560012 India

## Abstract

We report zero-field-cooled spontaneous-positive and field-cooled conventional-negative exchange bias effects in epitaxial bilayer composed of La_0.7_Sr_0.3_MnO_3_ (LSMO) with ferromagnetic (FM) and Eu_0.45_Sr_0.55_MnO_3_ (ESMO) with A-type antiferromagnetic (AF) heterostructures respectively. A temperature dependent magnetization study of LSMO/ESMO bilayers grown on SrTiO_3_ (001) manifest FM ordering (T_C_) of LSMO at ~320 K, charge/orbital ordering of ESMO at ~194 K and AF ordering (T_N_) of ESMO at ~150 K. The random field Ising model has demonstrated an interesting observation of inverse dependence of exchange bias effect on AF layer thickness due to the competition between FM-AF interface coupling and AF domain wall energy. The isothermally field induced unidirectional exchange anisotropy formed at the interface of FM-LSMO layer and the kinetically phase-arrested magnetic phase obtained from the metamagnetic AF-ESMO layer could be responsible for the spontaneous exchange bias effect. Importantly, no magnetic poling is needed, as necessary for the applications. The FM-AF interface exchange interaction has been ascribed to the AF coupling with $\sum {J}_{ex}\vec{{S}_{{\rm{FM}}}}\cdot \vec{{S}_{{\rm{AF}}}}$ (${J}_{ex}\approx {J}_{AF}$, coupling constant between AF spins) for the spontaneous positive hysteresis loop shift, and the field-cooled conventional exchange bias has been attributed to the ferromagnetically exchanged interface with ${J}_{ex}\approx {J}_{F}$ (coupling constant between FM spins).

## Introduction

A rich spectrum of exotic and unexpected properties have been unveiled at hybrid magnetic interfaces by controlling and understanding complex oxide heterostructures at the atomic level, providing a potential route to design modern day devices^1–5^. Such artificially constructed interfaces between two strongly correlated electron systems has garnered renewed research interest from the scientific community for their unusual properties and unprecedented physical phenomena resulting from charge transfer, magnetic frustration, and orbital reconstruction across the interfaces^6–11^. Depending on rare earth (RE = La, Pr, Sm, Gd and Eu) and A-site cations, manganese-based RE_1−*x*_A_*x*_MnO_3_ (A = Ca, Sr and Ba) perovskite systems exhibit double exchange and superexchange interactions between the adjacent Mn^3+^ and Mn^4+^ magnetic species. This leads to emergent magnetic phenomena such as half-metallic ferromagnetism, colossal magnetoresistance (CMR), ferromagnetism with insulating properties and spin-glass (SG) behaviour^12–14^. It has been shown that in charge-ordered manganites the magnetic field induced metamagnetic behavior from antiferromagnetic-insulator to ferromagnetic-metallic phase was assigned to the intrinsic phase separation, i.e., coexistence of competing magnetic phases in micro/nano length scales^15, 16^. Such a first-order magnetic irreversibility does not go back to the initial phase once the field is withdrawn, called as ‘kinetically phase-arrested’ magnetic phase^15, 16^, which is responsible for the CMR effect and other functional properties like, magnetoelectric and magnetocaloric effects^16–18^.

An interfacial phenomenon that has captured much research attention is the exchange bias (EB) effect^19, 20^ induced by quantum mechanical exchange coupling at the ferromagnetic (FM) and uncompensated antiferromagnetic (AF) interface. In the case of FM and compensated AF interface systems the extrinsic factors such as interface roughness and spin canting also contribute to the EB effect^21–23^. Some other models such as domain pinning and interface frozen provide required interface exchange coupling for the EB effect^24^. In proximity to an AF pinning layer, the FM phase can experience an exchange-induced unidirectional anisotropy after field-cooling (FC) the system through the AF Neel temperature (T_N_). This is manifested by a hysteresis loop shifting along the magnetic field axis quantified by an amount µ_0_H_EB_ (where µ_0_ is the vacuum permeability and H_EB_ is the exchange bias field). At present, there are efforts increasing to artificially construct heterointerfaces for the constant miniaturization of memory devices such as magnetic read heads and spin valves/tunnel junctions. These magnetic nanostructure devices have motivated researchers to investigate the EB effect with reduced lateral dimensions comparable to the AF domain size.

Recently, it has reported that some materials exhibit zero-field-cooled (ZFC) exchange bias effect; hysteresis loop shift has been noticed even without magnetic annealing. This is termed as spontaneous exchange bias (SEB) effect^25–28^. Here, the unidirectional anisotropy can be shaped during the isothermal virgin magnetization process, while in conventional exchange bias (CEB) systems such unidirectional anisotropy can be introduced only during the FC mode. Considering the applications, the SEB effect will be of great interest for electric-field control of EB devices as it eliminates the requirement of an external magnetic field to create unidirectional anisotropy^29^. Also, magnetic read heads with high sensitivity demand a tunable SEB effect. Previously, a small M(H) loop shift after zero-field cooling was considered as an experimental artifact. Later Saha *et al*., have provided a theoretical model for a small but intrinsic effect in Ni_80_Fe_20_/Ni_50_Mn_50_ system^30^ and experimentally observed a large SEB effect in Ni-Mn-In bulk alloy^27^. On the other hand, ZFC unidirectional anisotropy is induced by ‘super-interaction bias coupling’ between the FM core of Bi_2_Fe_4_O_9_ and the surface canted AF structure of BiFeO_3_ nanocomposite^26^. Later, Krishna *et al*. have reported the significance of magnetic frustration due to spin disorder and consequently observed SEB effect with a giant value of ~12 kOe in the polycrystalline La_1.5_Sr_0.5_CoMnO_6_ system^28^. It was realized that the mechanism for SEB is not generalized; rather it is a system dependent property. Therefore, elucidation of an unique driving mechanism to induce the hysteresis loop shift without magnetic annealing the thin films is currently an open issue in the development of spintronic devices. An experimental knowledge about the nature of coupling in FM/AF interfaces and the magnetic properties of AF layer upon which EB strongly depends is essential to understand this effect further. However, less attention has been paid to probe the role of AF layer thickness on EB effect, and no reports are available on the systematic study of SEB effect in AF/FM bilayer or multilayer heterostructures.

To investigate the aforementioned issues, perovskite-structure based manganites are particularly suitable. They are appropriate for the study of interface effects because of their rich phase diagram with a myriad of magnetic phases. As per the Hubbard model of strongly correlated electron systems, a reduction in the bandwidth of the *e*_*g*_ - electrons of Eu_1−x_Sr_*x*_MnO_3_ manganites (the quenched random disorder proportional to the Eu/Sr ratio) can yield a substantial change in the properties of the material^31^. For example, ferromagnetism with metallic behavior at 0.38 ≤ *x* ≤ 0.47 and SG phase with insulating state at 0.48 ≤ *x* ≤ 0.5 were observed. At higher doping of Sr (0.51 < *x* *<* 0.6), it is layered A-type antiferromagnetism with insulating behaviour; this changes to chained C-type antiferromagnetism for *x* ≥ 0.6. A strong competition was observed between A-type AF phase and charge/orbital ordering with a modulation vector of (0, *q*, 0) where *q* ~1/3 in Eu_1-*x*_Sr_*x*_MnO_3_ (*x* ≥ 0.5). Magnetic and electronic phases that are close to energy stability are sensitive to external magnetic fields^31^. A remarkable property of Eu_1−x_Sr_x_MnO_3_ metamagnetic systems is the field-induced magnetic phase transition, which can be employed to pin interface FM spins with neighbouring magnetic layers. In this regard, we have chosen Eu_0.45_Sr_0.55_MnO_3_ (ESMO) exhibiting A-type AF ordering (consisting of in-plane double-exchange mediated FM sheets coupled antiferromagnetically along the out-of-plane direction) and La_0.7_Sr_0.3_MnO_3_ (LSMO) as the typical FM layer to investigate the EB phenomena originating from interface exchange interaction. In this article for the first time, we report the rare coexistence of SEB and CEB effects in epitaxial bilayers consisting of ESMO and LSMO deposited on the SrTiO_3_ (STO) (001) substrate. Our experimental results not only open up a new path to realize the SEB effect and determine the nature of FM-AF interface exchange coupling but also provide a broad opportunity to tailor the SEB with AF layer thickness and temperature. It promotes the application of manganites in magnetic memory devices.

## Results and Discussion

**Figure 1:**
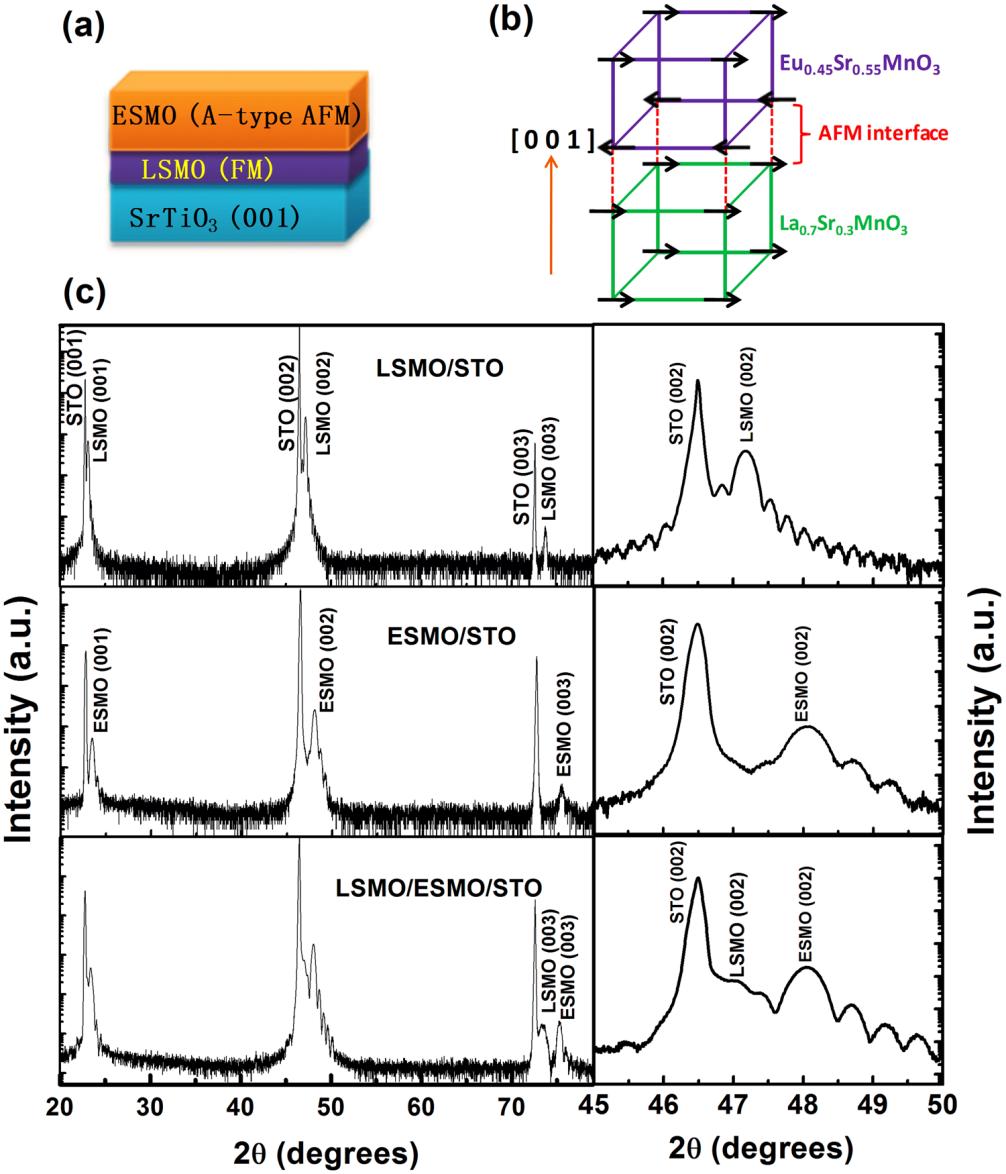
**(a)** Schematic representation of LSMO/ESMO bilayers heterostructure deposited on STO (001), **(b)** illustration of the lattice structure associated with simplified spin configurations in the FM-LSMO and A-type AF order in ESMO layers and the resultant interface AF arrangement, and **(c)** XRD-θ/2θ normal scan for the LSMO and ESMO single layers and LSMO/ESMO bilayer, right side to (**c**) indicates the enlarged view of XRD data around the (002) peak.

In Fig. 1(a), we present the schematic heterostructure of ESMO/LSMO bilayer deposited on STO (001) substrate. The illustration of the simplified magnetic order, as FM in LSMO bottom layer and layered A–type AF in top ESMO layer and the resultant interface AF spin arrangement, is shown in Fig. 1(b). In Table (1), we give the bulk lattice constants of LSMO (a_LSMO_ = 3.876 Å) and ESMO (a_ESMO_ = 3.8501Å) and the corresponding lattice mismatch of LSMO and ESMO films with respect to the STO substrate (a_STO_ = 3.905 Å). However in the present study, since the ESMO layer was grown on LSMO, its lattice mismatch with LSMO is significantly small. The XRD–θ/2θ pattern of (00 l) symmetric reflections of LSMO and ESMO single layers as well as bilayers on STO (001) has been measured (see Fig. 1(b)) to characterize the structural properties of as prepared films. The observation of only pseudo-cubic (00 l) peaks in LSMO and ESMO films demonstrates the out-of-plane epitaxy and good crystallinity with STO. A magnified view of θ/2θ scan around (002) reflection for the LSMO/ESMO bilayer and the single reference layers is shown at the right of Fig. 1(b). Well-defined Laue thickness interference fringes are clearly visible around (002) peak is another feature of homogenous and coherent crystal growth of films. Such thickness fringes are absent for the thicker ( > 60 nm) AF layers (not shown here) due to a decrease in high order peak intensity. As shown in the symmetric - θ/2θ scan around (002), the LSMO and ESMO peaks show a noticeable shift from their bulk lattice parameters (a_pseudocubic_ ~3.8760 Å for LSMO) and (a_pseudocubic_ ~3.8501 Å for ESMO) indicates that both LSMO and ESMO are highly strained films. The out-of-plane lattice parameters of LSMO and ESMO films and their lattice mismatch on STO are shown in Table (1) consistent with previous reports; the LSMO layer shows tensile strain due to its coherent growth on STO^32^. As shown in Table (1), the *c*-axis lattice parameter of the ESMO layer (~3.78 Å) is smaller than its pseudocubic lattice constant (~3.85 Å) suggesting that the ESMO was also grown under tensile strain.

**Table 1:** List of in-plane (*a*), out-of-plane (*c*) lattice parameters and inter-plane spacing (*d*) and lattice mismatch of ESMO/LSMO bilayers and single LSMO and ESMO references layers on STO obtained from the asymmetric RSM data along (103) reflection.

layer	Lattice mismatch	*d*-value (Å)	*d*-parameter mismatch	In-plane lattice parameter (*a*) Å	Film lattice parameter mismatch	Out-of-plane lattice parameter (*c*) Å and mismatch
**ESMO/LSMO/STO along (103)**						
STO		1.2348		3.9050		3.9050
LSMO (bulk *a* = 3.876 Å)	0.7%	1.2226	0.955% (w.r.to STO)	3.9127	−0.197%	3.8610 and 1.12%
ESMO (bulk *a* = 3.8501 Å)	1.4%	1.1986	1.9% (w.r.to LSMO) 2.9% (w.r.to STO)	3.8556	1.48% (w.r.to LSMO) 1.26% (w.r.to STO)	3.7832 and 3.1%
**LSMO/STO along (103)**						
LSMO	0.7%	1.2199	1.2%	3.910	−0.127%	3.8521 and 1.3%
**ESMO/STO along (103)**						
ESMO	1.4%	1.2006	2.7%	3.9253	−0.51%	3.7832 and 3.1%

**Figure 2:**
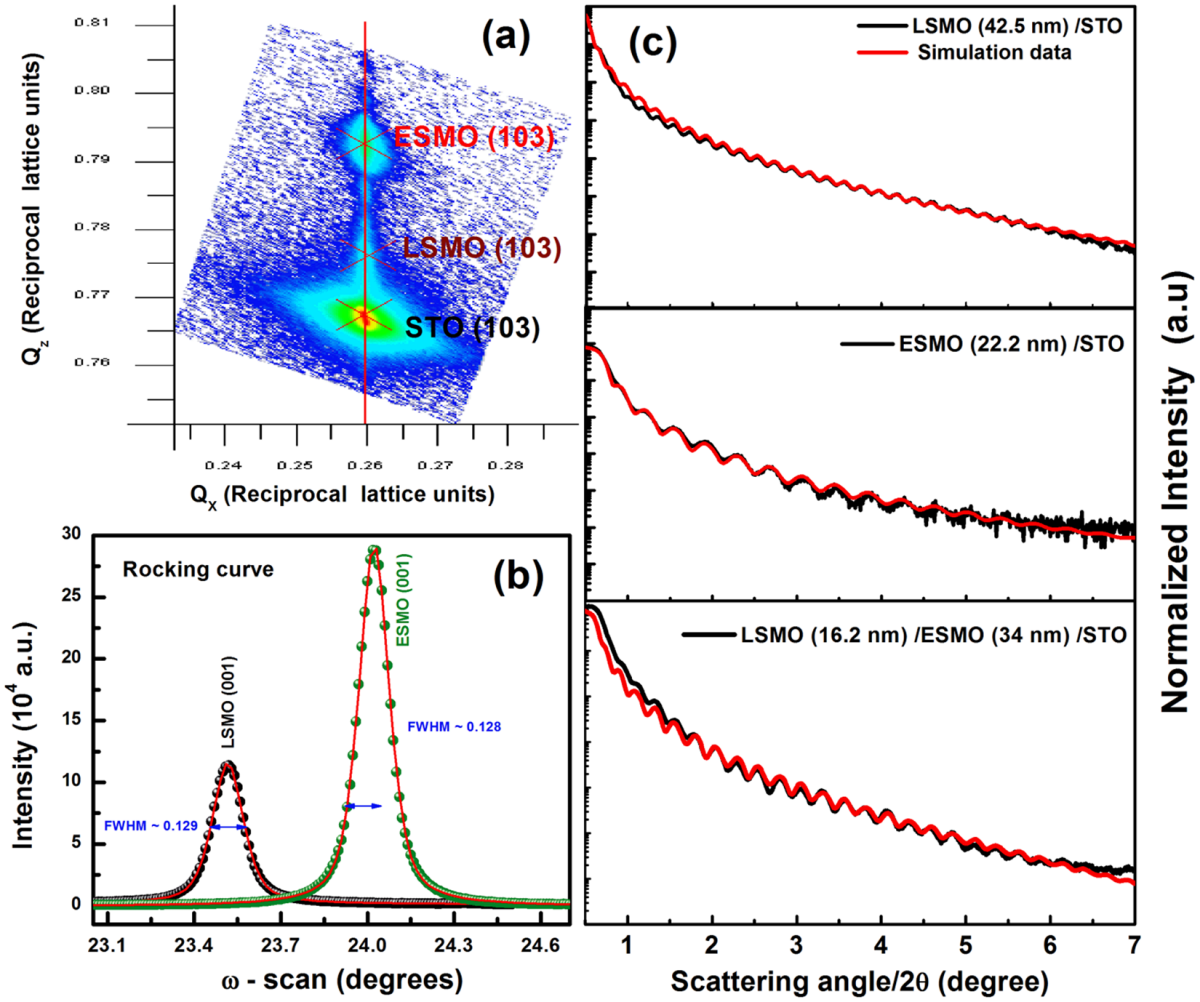
(**a**) Asymmetric RSM data along the (103) peak and (**b**) rocking curve for LSMO/ESMO bilayers and (**c**) XRR for the single LSMO and ESMO reference layers and bilayer.

To further confirm the coherent state of LSMO/ESMO bilayer, an asymmetric RSM scan around (103) Bragg reflection and a rocking curve (ω - scan) have been recorded as shown in Fig. 2(a) and (b) respectively. The RSM data clearly illustrates that the films are fully strained. The peaks corresponding to the films and the substrate appear for the same value of in-plane (Q_x_) position demonstrating the epitaxial nature of as-prepared samples. Multiple spots observed at the STO region can be attributed to the presence of several small crystallite blocks corresponding to the different substrate reflections^33^. On the other hand, the full widths at half maximum (FWHM) of the rocking curves at (002) in the bilayers is low (in the range of ~0.1°) suggesting a good crystalline order and the absence of structural defects. In case of individual reference layers, this FWHM value is ~0.17° for ESMO and 0.07° for LSMO on STO (see in the supplementary information (SI), Figure S1). To measure the thickness of the ESMO (~34 nm)/LSMO (~16.2 nm) bilayers and the single LSMO (~44.5 nm) and ESMO (~22.2 nm) reference layers, we employed the typical XRR measurement; results are shown in the Fig. 2(c). XRR pattern was simulated well in the model with uniform scattering length density throughout the film and an abrupt film-substrate interface using Rigaku’s Global fit program. The observed Kiessig fringes (even up to 2θ ~7°) and the average root mean square roughness of the order of ~0.2–0.45 nm clearly suggest that LSMO and ESMO were prepared on STO as layer-by-layer growth with sharp and smooth interfaces. From the fitting the obtained volume data for LSMO is ~3.82 × 10^−7^ cm^3^, ESMO is ~4.08 × 10^−7^ cm^3^ and LSMO/ESMO is ~6.02 × 10^−7^ cm^3^.

**Figure 3:**
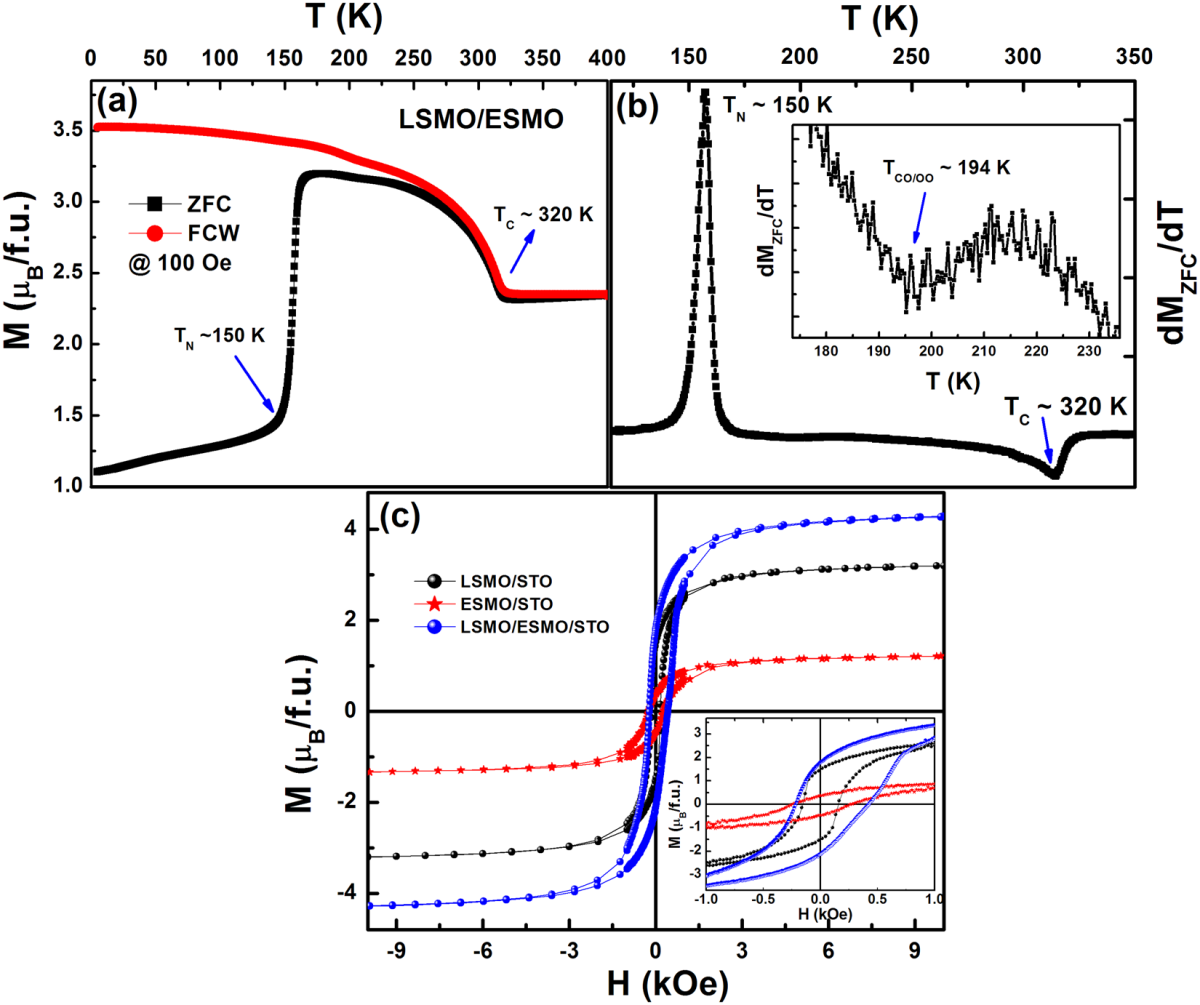
(**a**) M vs T (K) data for the LSMO/ESMO bilayer in the ZFC and FC protocols, (**b**) first derivative of ZFC-M with respect to T (K), to represents the various magnetic transitions, and its inset is the magnified view of the dM_ZFC_/dT vs. T (K) to show the charge order/orbital ordering at ~194 K. (**c**) Isothermal M(H) curves at 5 K for the single reference layers and bilayer (inset is the enlarged view of M vs. H at lower fields).

FM ordering (T_C_) of the LSMO single layer (see SI, Figure S2) obtained from the first derivative of magnetization is ~330 K; matches well to the previously reported LSMO/STO epitaxial thin films^34^. The magnetic signal of ESMO thin film (not shown here) is weak due to its AF nature, similar to the SrMnO_3_ in LSMO/SrMnO_3_ bilayers^14^. The Fig. 3(a) shows the temperature variation of ZFC and FC magnetization for bilayers at 100 Oe; FM-T_C_ corresponding to LSMO is ~320 K and charge-ordering (CO)/orbital-ordering (OO) transition of Mn^3+^ and Mn^4+^ ions corresponding to ESMO emerges at ~194 K with modulation vector (0, *q*, 0) where *q* ~1/3 in the *Pbnm* space group^35^. Further, a sharp fall in M_ZFC_ on decreasing in temperature to ~150 K matches well with AF ordering (T_N_) of the ESMO layer, consistent with the magnetic phase diagram of ESMO single crystals^31^. Here, it is to be noted that the observation of long-range CO/OO at ~194 K (as shown in the first derivative of ZFC magnetization, Fig. 3(b)) in the bilayer strongly suggests that the ESMO layer was deposited with the prerequisite stoichiometry of Sr = 0.55; for low doping of Sr (0.47 < *x* < 0.5), the ESMO-layer does not exhibit such long-range CO/OO ordering.

Isothermal field-dependent magnetization, M(H) loops for LSMO/ESMO bilayer and single ESMO and LSMO reference layers, are shown in Fig. 3(c). All the samples exhibit the hysteresis loop at 5 K with different coercive fields (H_C_) and saturation magnetization (M_S_) and remnant magnetization (M_R_) values. The complex magnetic phases, i.e., A-type AF and isothermally field induced FM phase like behaviour in ESMO layers are explained in a later section. The low temperature spin-only contribution to M_S_ can be theoretically estimated^14^ as the total contributions expected from the *x*Mn^3+^ and (1 − *x*) Mn^4+^ ions as,

1 $${M}_{S}=x4{\mu }_{B}+(1-x)3{\mu }_{B}=(3+x){\mu }_{B}$$

2 $${M}_{S}=x4{\mu }_{B}-(1-x)3{\mu }_{B}=(7x-3){\mu }_{B}$$

The above eq. (1) is applicable to the FM alignment of Mn^3+^/Mn^4+^ species in LSMO layer and eq. (2) can be used to explain the AF alignment of Mn^3+^/Mn^4+^ in ESMO layer. Accordingly, the calculated *M*_S_ for LSMO layer is ~3.22 µ_B_/Mn and ~0.91 µ_B_/Mn for the ESMO layer; they are good in agreement with the measured values at 5 K for 6 kOe are ~3.3µ_B_/Mn and ~1.3 µ_B_/Mn respectively. The H_C_ value increases significantly from ~160 Oe for the single LSMO layer to ~325 Oe for the LSMO/ESMO bilayer. The M_R_ ( = (M_R+_ − M_R−_)/2) shows a nominal increase from ~1.54 µ_B_/Mn for the LSMO layer to ~1.97 µ_B_/Mn for the bilayer. Though the single ESMO layer shows small magnetization values, its H_C_ (~245 Oe) and M_R_ (~0.23 µ_B_/Mn) are in accordance with literature data^31^ and prepared ESMO bulk samples (see SI, Figure S(3)). In bilayer, the total magnetization is the sum of individual LSMO and ESMO reference layers, as confirmed from the Fig. 3. This suggests that the ESMO magnetic phase does not disrupt the LSMO-FM ordering, which excludes the possibility of SG phase presence at the LSMO-ESMO interface. A linear variation of the virgin curve magnetization with a stair-like change at 2.4 T and a no- saturation trend even up to 90 kOe are observed in the bulk ESMO polycrystalline sample; this indicates its strong AF behaviour (see SI, Figure S(3)). Further features such as irreversibility, the absence of metamagnetic beahviour for the consequent field sweeps and the virgin curve falling outside the M(H) hysteresis envelope indicate that such an isothermally field-induced magnetic phase transition is of the first-order kind^31, 35, 36^. For low doping of Sr ≤ 0.5; there are clear field induced sharp magnetization jumps for fields ≤ 50 kOe (not shown). The ESMO layer for Sr = 0.55 has a larger volume fraction of AF phase, which requires high measuring fields (>90 kOe) to employ the field-induced phase transition. Such a sharp rise in magnetization and a low field saturation tendency in the LSMO single layer indicates its typical FM nature. On the other hand, a low field linear variation of magnetization and a non-saturation tendency (even for 6 kOe) suggests the presence of AF spin correlations in the ESMO single layer as well as in the bilayer (see the normalized virgin curve M(H) loop at 5 K, in Figure S3(b) of SI).

**Figure 4:**
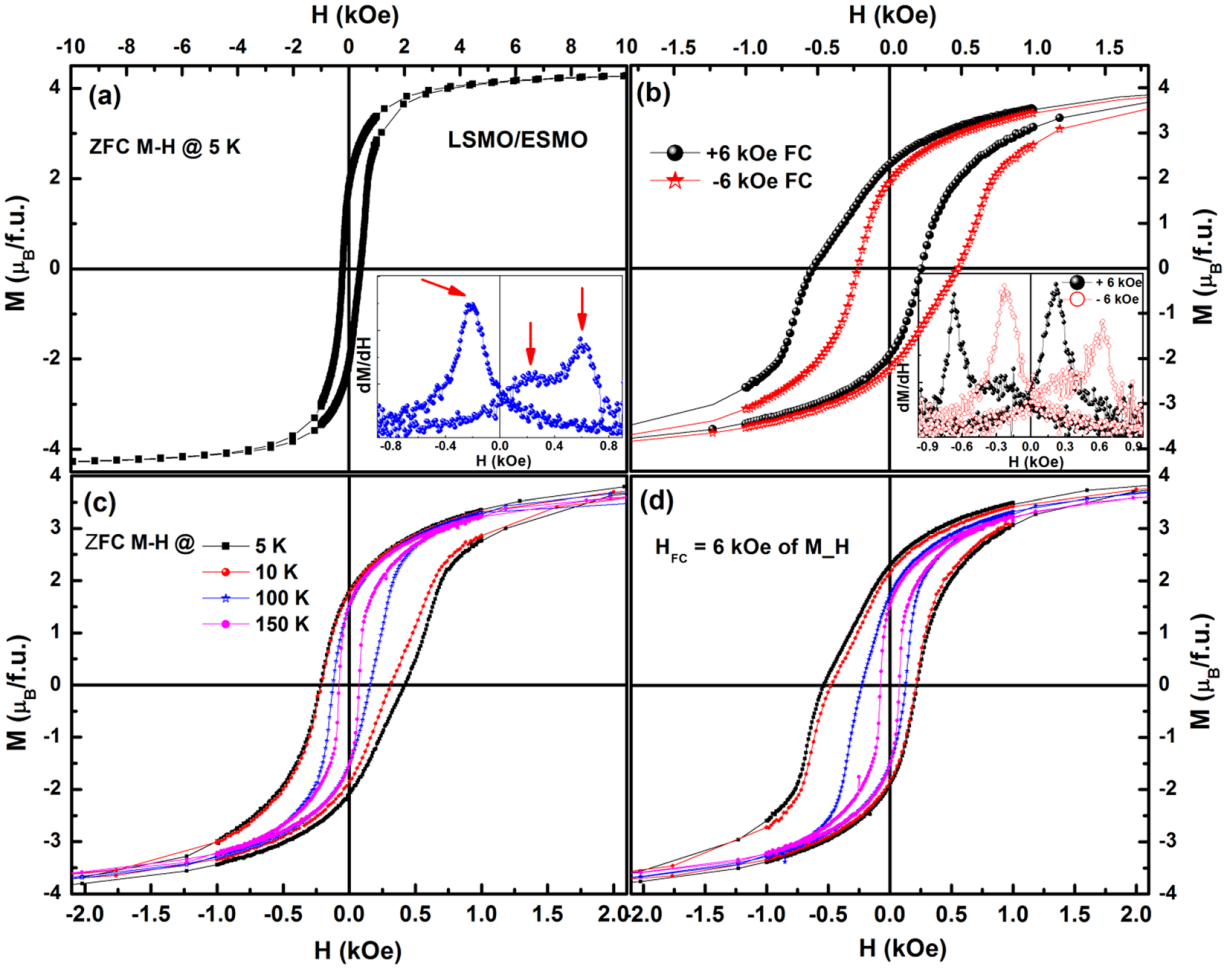
M(H) loops measured at 5 K in ZFC (**a**) and FC (**b**) modes for the LSMO/ESMO bilayers at 5 K, and their inset shows the dM/dH vs. H plots. (**c**,**d**) Is the isothermal M(H) loops at different temperatures for the ZFC and FC modes, respectively. For the clarity, only the data between −10 kOe to 10 kOe are shown in the figures, while the actual measurements took place in between −40 kOe to 40 kOe.

As shown in Fig. 4(a), the bilayer exhibits the asymmetric magnetization loop at 5 K after ZFC, i.e., an obvious loop shift towards the positive field axis, which is a characteristic feature of EB effect. To confirm this loop shift as a bilayer feature, we measured the M(H) loops for single LSMO and ESMO reference layers; they are symmetric about the origin as shown in the inset of Fig. 3(c). Further, the loop shift was predominantly represented by plotting dM/dH vs H data (Fig. 4(a)). It may be seen that in the ZFC case the first reversal magnetization (i.e., in the negative field sweep) shows a sharp single peak with low value of H_C_. While the second reversal magnetization (in the positive field sweep) exhibits two peaks corresponding to the LSMO at low fields and the ESMO magnetization switching at high fields, resulting in the positive exchange bias (PEB) effect. Such a ZFC asymmetry in the M(H) loop shift is termed as SEB effect^25, 26^. Here, the H_C_ enhancement towards the positive field could be attributed to the pinning strength of the AF domains exerted on the neighbouring FM spins during magnetization reversal of the FM layer. Now with the bilayer biased with H_FC_ = +6 kOe from 400 K down to 5 K, the loop peaks are reversed and yield to the transformation towards the negative field axis (see inset in Fig. 4(b)); this shift is denoted as CEB effect. Such a CEB was further confirmed by measuring the M(H) loop after cooling with H_CF_ = −6 kOe (Fig. 4(b). It can be seen that the M(H) loop with H_FC_ = + 6 kOe is symmetrically opposite to that with H_FC_ = −6 kOe. This confirms the reproducible EB shift, after performing arbitrary measurements from several subsequent runs with different field ramping rates and measuring fields. To rule out the experimental artifacts further, we carried out a control measurement at 5 K on single LSMO and ESMO layers with FC-M(H) loops and found the absence of CEB in both (see SI, Figure S4). This evidently suggests that bilayer structure with considerable interface strain play a significant role in the exchange coupling and the loop shift can be intrinsically originated from the interface magnetic coupling. Therefore, the observed ZFC-PEB and FC-CEB effects indicate that the spin interface structure can be subtle to the magnetic history of the sample.

**Figure 5:**
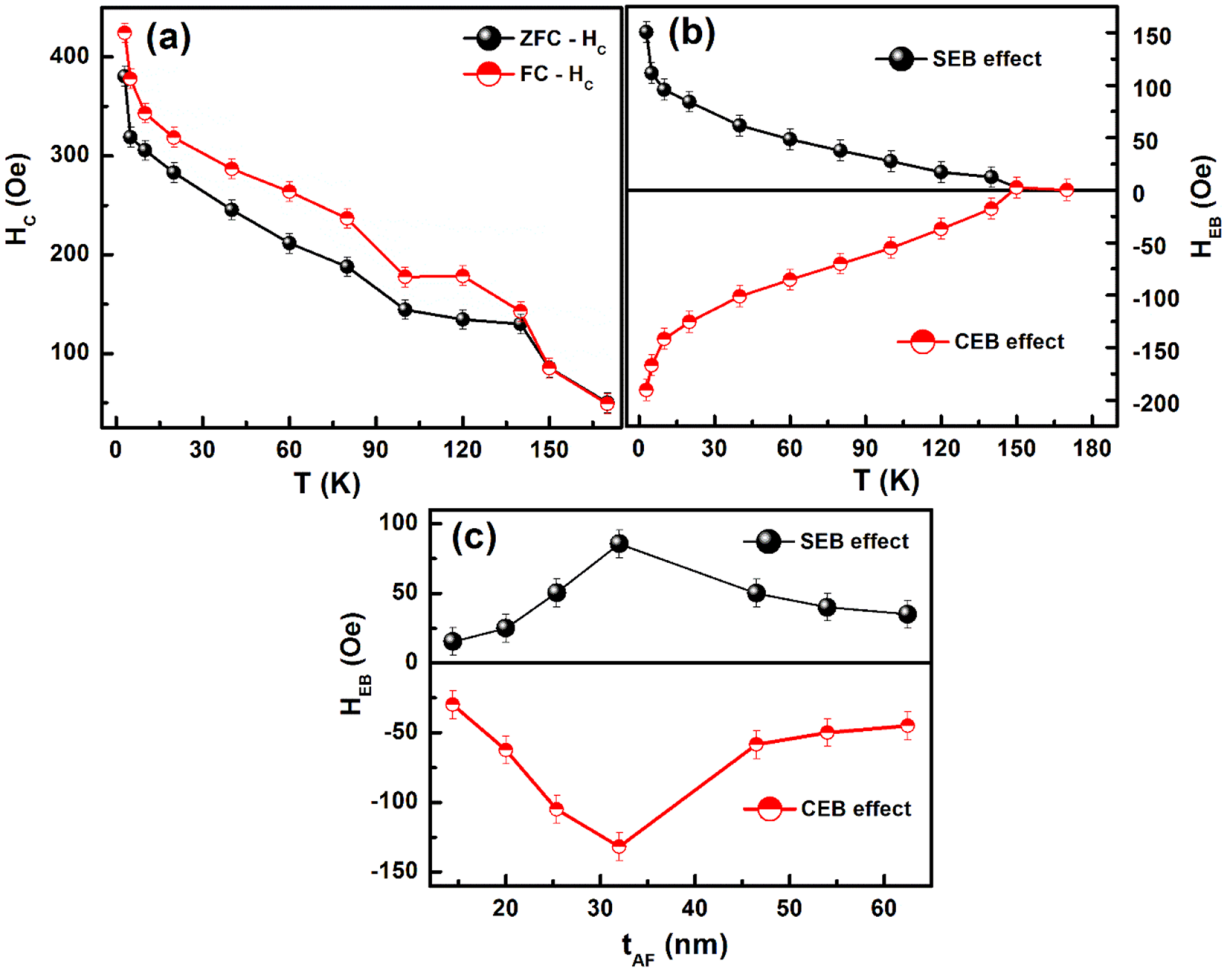
**(a**,**b)** Represents the temperature dependence of H_C_ and H_EB_ both in ZFC and FC modes, respectively. And **(c)** is the H_SEB_ and H_CEB_ as a function of AF layer thickness (t_AF_) at 10 K. Lines are to guide the eye.

Figure 4(c) and (d) shows isothermal M(H) loops for ZFC and FC protocols, respectively to investigate the temperature-dependent H_C_ and the EB effect. Here, the loop asymmetry can be quantified as the EB field; H_EB_ = (H_C1_ − H_C2_)/2 (where H_C1_ and H_C2_ are the positive and negative coercive fields respectively). The estimated loop shifts at 5 K in the ESMO (~32 nm) /LSMO (~16 nm) bilayer are H_EB_ = +86 Oe and −135 Oe which translate as the PEB in ZFC mode and negative exchange bias in FC mode, respectively. The asymmetry of the ZFC and FC M(H) loops decreases with increasing temperature and vanishes at T_N_ ~150 K. The temperature dependence of H_C_ = (H_C1_ + H_C2_)/2 is obtained in both the ZFC and FC modes (shown in Fig. 5(a)); it shows an increasing trend below T_C_ ~320 K and shows an anomaly at T_N_ ~150 K, indicating that an additional source of domain wall pinning effect in the FM layer exists. Below T_N_, FC-H_C_ is larger than ZFC-H_C_. On the other hand, the appearance of both H_SEB_ and H_CEB_ effects (Fig. 5(b)) below the advent of AF ordering and the absence of signatures of EB effect above T_N_ strongly indicate that interface exchange coupling originates only below T_N_. On further decrease in temperature, EB starts to increase linearly up to 10 K followed by a sharp rise at low temperatures. It is important to note that the absence of exponential decay of H_C_ and EB effect with temperature strongly excludes the possibility of interface SG originated EB effect in present bilayer system^14, 37^.

Further, we clarify the AF layer thickness (t_AF_) dependence of interface exchange coupling. The H_SEB_ and H_CEB_ variation with t_AF_ for the LSMO/ESMO bilayer is represented in Fig. 5(c). As per the first theoretical model proposed by Meiklejohn-Bean (MB)^38, 39^, the AF layer should possess the sufficient anisotropic energy to preserve the spins from the magnetization reversal process caused by the FM/AF interface exchange interaction. Accordingly, the loop shift (H_CEB_ or H_SEB_) occurs only above a certain critical AF layer thickness (t^cr^_AF_); ${K}_{AF}{{t}^{cr}}_{AF}\ge {J}_{INT}$ where *K*_*AF*_ is the anisotropy constant of the AF layer. In the present study, the ${{t}^{cr}}_{AF}$ value obtained at 5 K for the LSMO/ESMO bilayers is ~12 nm; EB increases with t_AF_ and shows a maximum shift at t_AF_ ~32 nm. Further, there is a decreasing trend for the thicker AF layers. Such an AF layer thickness dependent peak in the EB effect was observed as in IrMn/Co films^40, 41^. Binek *et al*. have generalized the MB model and obtained a relation to demonstrate the increase of EB effect with AF layer thickness^42, 43^. However, this model does not explain the inverse proportion of H_EB_ on t_AF_ and the nature of FM/AF interface exchange coupling. Later, Malozemoff proposed a random field Ising model at the FM/AF interface, which can demonstrate the inverse dependence of H_EB_ on t_AF_^20^. It was assumed that in the case of AF layer having larger thickness, the presence of random exchange interactions between FM and AF spins could create the local energy minima and effectively pin the AF layer domain wall. Accordingly, the AF lattice breaks into domains of size that can be estimated from the competition between FM-AF interface exchange coupling and domain wall (DW) energy. The interfacial (INT) exchange energy per unit area is

3 $${E}_{INT}=\frac{-{J}_{FM \mbox{-} AF}}{r{D}_{AF}}$$

where, *J*_*FM*-*AF*_ is the FM-AF interface exchange coupling constant, *r* is the AF spin distance and *D*_*AF*_ is the AF domain size. The DW energy per unit area of the AF layer with exchange constant *J*_*AF*_ can be written as,

4 $${E}_{DW,AF}=\frac{{\pi }^{2}{J}_{AF}}{4r{D}_{AF}}$$

Further, the equilibrium domain size can be obtained by minimizing the total interfacial energy per unit FM-AF interface area, i.e., *E*_*INT*_ + *E*_*DW*,*AF*_ = 0 Therefore,

5 $${D}_{AF}=\frac{{\pi }^{3}{J}_{AF}{t}_{AF}}{4{J}_{AF \mbox{-} FM}}$$

From the Eqs (4) and (5), the evaluated expression for the interface exchange energy^20^ [36] is,

6 $${E}_{INT}=\frac{-{({J}_{FM \mbox{-} AF})}^{2}}{r{\pi }^{3}{J}_{AF}{t}_{AF}}$$

The above eq. (6) describes the inverse proportional relation of *E*_*INT*_ with t_AF_, which can explain the decrease in both H_CEB_ and H_SEB_ effects (as shown in the Fig. 5(c)) with an increase in AF layer thickness above t_AF_ ~32 nm. The loop shift dependence on t_AF_ may be trivial for larger thicknesses of the AF layer.

**Figure 6:**
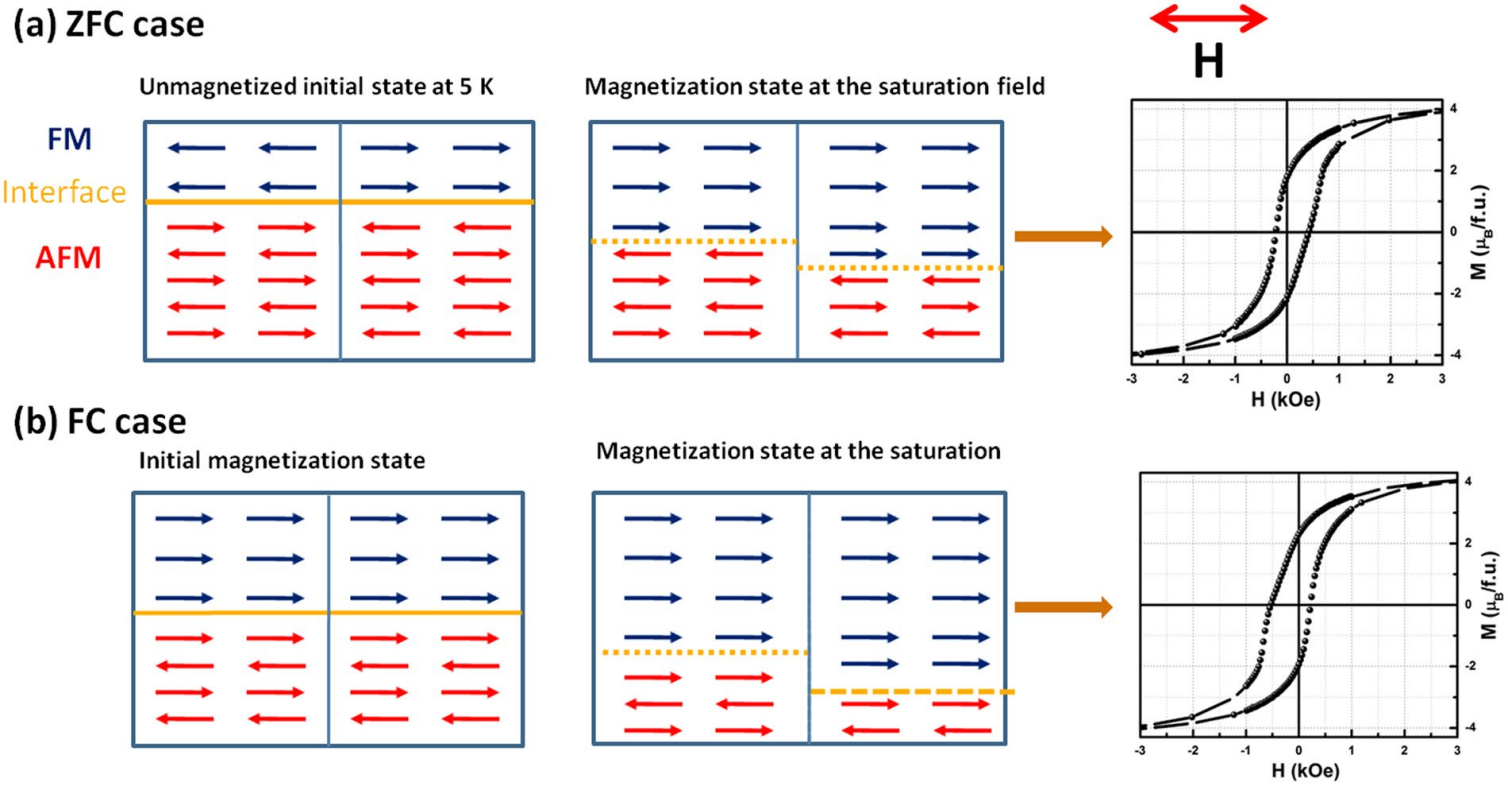
Schematic representation of the spin configuration at different stages (left): for the ZFC **(a)**, and FC cases **(b)**. Here note that the spin configuration is a simple cartoon to illustrate the interface coupling. The resultant hysteresis loop of SEB and CEB effects (right).

The observed ZFC-PEB and FC-CEB effects in the ESMO/LSMO bilayers can be illustrated based on the spin bidomain model with a variable FM-AF interface. Since the bilayer was cooled in the ZFC process, from an unmagnetized FM state to T < T_N_, the net magnetization of the FM and AF domains is zero due to their random alignment. In this case, the spin bidomain model can simplify the resultant magnetic structure, as two FM and AF domains parallel to the applied field with opposite directions. Figure 6(a) and (b) show simple cartoons that have been used to describe the variation in the isothermally field induced spin configuration at the initial and final magnetization states after the ZFC and FC processes. A qualitative appreciation can be drawn from these schematic figures in ZFC case during the initial magnetization process, FM domains grow at the cost of AF domains (i.e., the AF spins next to the FM layer align ferromagnetically) due to the field induced partial phase transformation of A-type AF to FM in the ESMO layer. After the removal of the measuring field, i.e., in zero-field state the converted FM state is kinetically phase arrested and form the extended spin pinning interface, resulting in a stable magnetic phase with unidirectional anisotropy. Then the exchange interaction at the interface breaks the symmetry and causes one of the AF sublattices to couple with an FM phase, assuming that the resultant interface spins are antiferromagnetically exchanged. Such a newly formed interface is fixed during the subsequent hysteresis loops; assumption made by Wang *et al*.^27^. A microscopic torque exerted by the AF pinning force on the FM layer leads to the unidirectional anisotropy. The measuring field $H={H}_{C}+|{H}_{E}|$ is required to overcome the interface AF exchange coupling and is the cause for the magnetization reversal. The resultant bilayer hysteresis loop shifts towards the positive field axis and is responsible for PEB (i.e., H_E_ > 0)^33, 44^. On the other hand in the FC case, as Tomioka *et al*. have investigated, the external FC can eliminate the CO/OO state and is the cause for the insulator to metal transition^31^. As a result of its CMR behaviour, the Mn^3+^-Mn^4+^ FM-double-exchange interactions eventually overcomes the localized AF superexchange interactions. Therefore, the resultant spin arrangement after FC has a non-zero magnetization value at 5 K and the AF spins polarize opposite to the ZFC case as shown in Fig. 6(b). Further, the isothermal field ramping induces more such FM clusters at the cost of the AF phase, the subsequent interface exchange interaction becomes FM, similar to the CEB systems. Then the measuring field $H=-{H}_{C}-|{H}_{E}|$ is required to overcome such FM interface interactions; the resulting bilayer hysteresis loop shifts towards the negative magnetic fields axis, i.e., the CEB effect as shown at the right in Fig. 6(b).

## Conclusions

In summary, we fabricated ESMO layers epitaxially conjoined at the interface to LSMO layers with a bilayer heterostructure form and also their single reference layers on SrTiO_3_ (001) substrate. We observed zero-field-cooled positive hysteresis loop shift and field-cooled negative hysteresis loop shift in the LSMO/ESMO bilayers below the T_N_ of ESMO ~150 K. And investigated the temperature and AF layer thickness dependence of spontaneous and conventional EB effects. It was found that isothermally field induced newly formed magnetic phases directly influence LSMO/ESMO interface interactions and create the pinned and uncompensated spins required for the EB effect. An experimental observation of the initial rise of H_CEB_ and H_SEB_ effects with AF layer thickness (t_AF_) are explained with the generalized Meiklejohn-Bean model and the inverse dependence of H_EB_ on t_AF_ (for t_AF_ > 32 nm) has been demonstrated with the Random field Ising model. In the observed positive exchange bias effect, zero-field-cooled magnetic interface coupling ($\sum {J}_{ex}\vec{{S}_{F}}\cdot \vec{{S}_{AF}}$) favours an AF alignment with ${J}_{ex}\approx {J}_{AF}$(coupling constant between AF spins) while it favours an FM alignment with ${J}_{ex}\approx {J}_{F}$(coupling constant between FM spins) in the field-cooled case and is responsible for conventional exchange bias. The conjunction of spontaneous exchange bias effect along with conventional exchange bias effect is a highly desirable attribute as it can reveal an additional degree of freedom that can be harnessed in spintronic device applications. Our observations offer a new perspective to study the EB effect without magnetically annealing the sample. Advancements in crafting the interfacial coupling with suitable bilayer combinations calls for technological breakthroughs, to explore future magnetic materials at ambient temperatures. Further x-ray absorption spectra (XAS) and x-ray magnetic circular dichroism (XMCD) spectroscopic studies on LSMO/ESMO bilayer samples are required to investigate the magnetic coupling and associated electronic orbital reconstruction at the interface.

## Experimental details

### Sample Fabrication

Polycrystalline LSMO and ESMO bulk samples were prepared by the solid-state reaction method. High purity (99.99%, Sigma-Aldrich) Eu_2_O_3_, La_2_O_3_, SrO and MnO_2_ materials were weighted as per the stoichiometry ratio. To achieve the high composition and homogeneous the samples were mixed thoroughly, heated to 1000 °C and then to 1300 °C with intermediate grindings until the desired single phase was obtained. Then obtained powder was pelletized with a dimension of 1″ target using 6–10 Mpa external pressure and sintered at 1350 °C for 24 hours. A bilayer heterostructure with LSMO as the bottom layer and ESMO as the top layer grew on STO (001) by using pulsed laser deposition (PLD). The epitaxial thin films were optimized by focusing KrF excimer laser (λ = 248 nm) with 3 Hz pulses and energy density of ~1.5–2 J/cm^2^ onto targets with ~200 mTorr of O_2_ background pressure while maintaining the substrate temperature at ~750 °C. After the deposition process, the films were annealed *in-situ* for 1 hour at the same temperature with 1 bar of O_2_ pressure and subsequently cooled down to the ambient temperature with a cooling rate of 5 °C/min. For reference, LSMO and ESMO single layers also deposited on STO with identical deposition conditions to study their individual properties.

### Structural and Magnetic Characterization

The structural characterization of the prepared bilayer was done by x-ray diffraction (XRD) with normal θ/2θ scan along the pseudo-cubic (00 l) reflections using Rigaku Smart Lab diffractometer. The same instrument was used to obtain the reciprocal space map (RSM) around (103) reflection. Film thickness was estimated using small angle x-ray reflectivity (XRR) measurement. The thermomagnetic and isothermal field dependent magnetization measurements were carried out in a commercial SQUID magnetometer. For the temperature dependent magnetization measurements under field-cooled (FC) and ZFC protocols, the samples were cooled from 400 K down to 5 K with and without applied magnetic fields respectively. The FC-M(H) loop measurements were done after cooling the samples in 6 kOe field from 400 K to the reference temperature; the field was applied along the in–plane (100) substrate direction (parallel to the sample edge). To evaluate the magnetization of prepared the films, virgin substrate data was also measured and the linear contribution from the diamagnetic STO was subtracted from the experimental data.

## Electronic supplementary material

Supplementary Information
