# Author Correction: Interface-induced spontaneous positive and conventional negative exchange bias effects in bilayer La_0.7_Sr_0.3_MnO_3_/Eu_0.45_ Sr_0.55_ MnO_3_ heterostructures

**DOI:** 10.1038/s41598-018-25730-z

**Published:** 2018-05-15

**Authors:** J. Krishna Murthy, P. S. Anil Kumar

**Affiliations:** Department of Physics, Indian Institute of Science, Bengaluru, 560012 India

Correction to: *Scientific Reports* 10.1038/s41598-017-07033-x, published online 31 July 2017

In Figure 3a, the y-axis ‘M (arbitrary units)’ is incorrectly given as ‘M (µ_B_/f.u.)’. The correct Figure 3a appears below as Figure 1.

**Figure 1:**
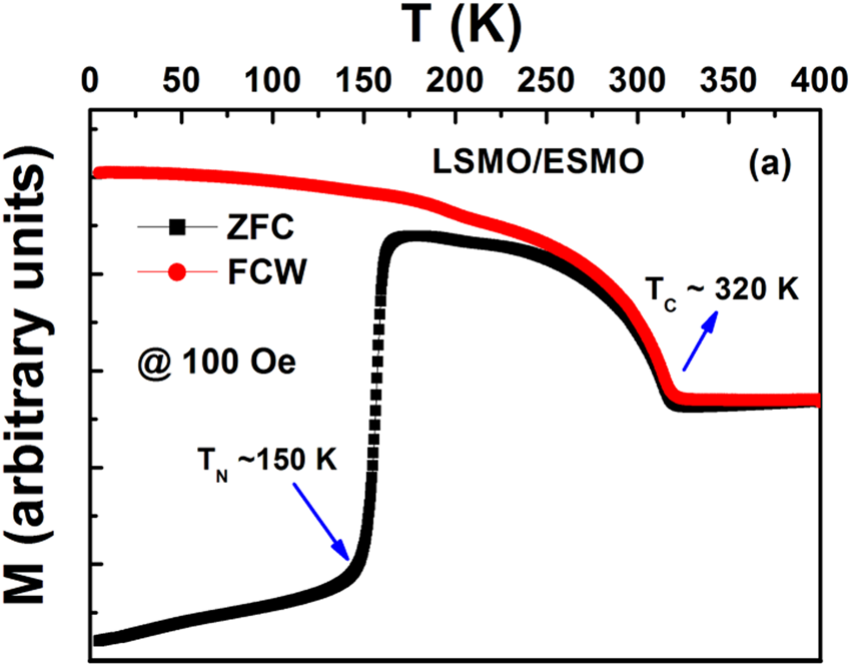
(**a**) M vs T (K) data for the LSMO/ESMO bilayer in the ZFC and FC protocols, (**b**) first derivative of ZFC-M with respect to T (K), to represents the various magnetic transitions, and its inset is the magnified view of the dM_ZFC_/dT vs. T (K) to show the charge order/orbital ordering at ~194 K. (**c**) Isothermal M(H) curves at 5 K for the single reference layers and bilayer (inset is the enlarged view of M vs. H at lower fields).

