# Supplementary material for: Interface-induced spontaneous positive and conventional negative exchange bias effects in bilayer La0.7Sr0.3MnO3/Eu0.45Sr0.55MnO3 heterostructures
Source: Sci Rep. 2017 Jul 31;7:6919. doi: 10.1038/s41598-017-07033-x (PMC5537235; doi:10.1038/s41598-017-07033-x)
Supplement: Supplementary file 1 — Supplementary Information [file 41598_2017_7033_MOESM1_ESM.pdf]

Supplementary Information

Interface induced spontaneous positive and conventional negative exchange bias effects in bilayer $La_{0.7}Sr_{0.3}MnO_3$/$Eu_{0.45}Sr_{0.55}MnO_3$heterostructures

J Krishna Murthy and P S Anil Kumar

Department of Physics, Indian Institute of Science, Bengaluru, India-560012

ABSTRACT

We report zero-field-cooled spontaneous-positive and field-cooled conventional-negative exchange bias effects in epitaxial bilayer composed of $La_{0.7}Sr_{0.3}MnO_3$ (LSMO) with ferromagnetic (FM) and $Eu_{0.45}Sr_{0.55}MnO_3$ (ESMO) with A-type antiferromagnetic (AF) heterostructures respectively. A temperature dependent magnetization study of LSMO/ESMO bilayers grown on $SrTiO_3$ (001) manifest FM ordering (T_C) of LSMO at ~320 K, charge/orbital ordering of ESMO at ~194 K and AF ordering (T_N) of ESMO at ~150 K. The random field Ising model has demonstrated an interesting observation of inverse dependence of exchange bias effect on AF layer thickness due to the competition between FM-AF interface coupling and AF domain wall energy. The isothermally field induced unidirectional exchange anisotropy formed at the interface of FM-LSMO layer and the kinetically phase-arrested magnetic phase obtained from the metamagnetic AF-ESMO layer could be responsible for the spontaneous exchange bias effect. Importantly, no magnetic poling is needed, as necessary for the applications. The FM-AF interface exchange interaction has been ascribed to the AF coupling with $\sum J_{ex} \vec{S}_{\mathrm{FM}} . \vec{S}_{\mathrm{AF}}$ ($J_{ex} \approx J_{AF}$, coupling constant between AF spins) for the spontaneous positive hysteresis loop shift, and the field-cooled conventional exchange bias has been attributed to the ferromagnetically exchanged interface with $J_{ex} \approx J_F$ (coupling constant between FM spins).

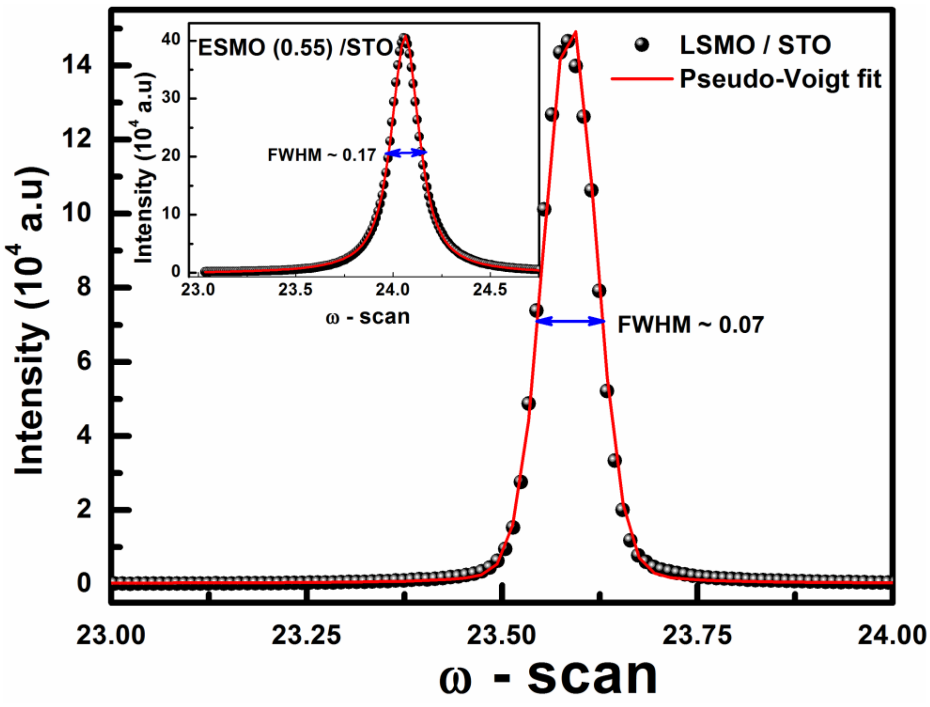

Figure S1: Rocking curves (ω - scan) of single LSMO and ESMO (inset) reference layers deposited on STO (001).

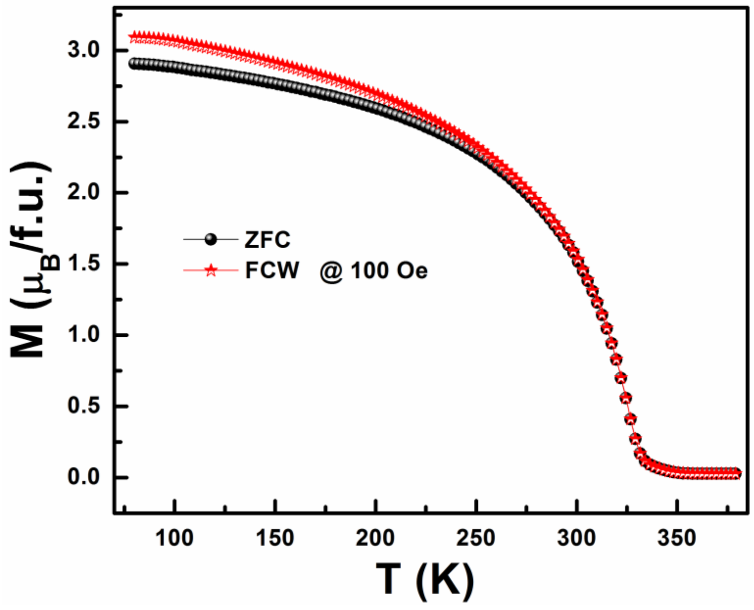

Figure S2: M (T) data of LSMO/STO sample under ZFC and FCW process for 100 Oe.

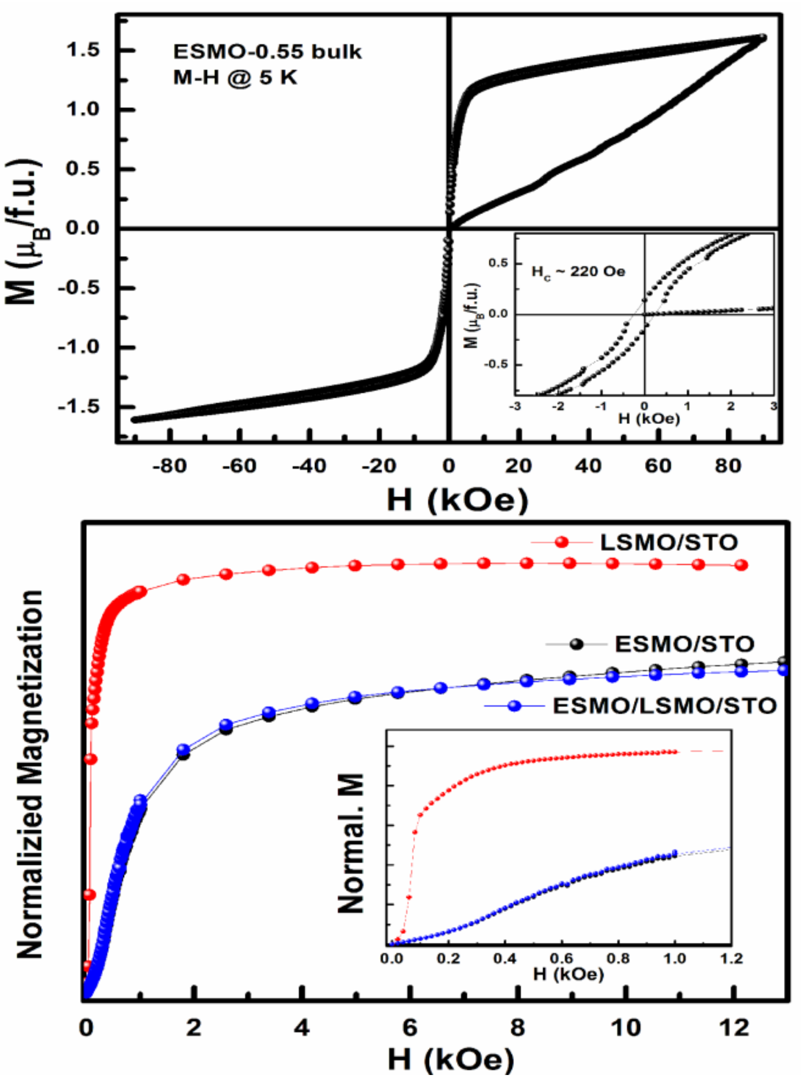

Figure S3: (a) M-H hysteresis loop of bulk $Eu_{0.45}Sr_{0.55}MnO_3$ polycrystalline sample, inset is the zoomed-in data of loop at low range of fields, and (b) isothermal virgin normalized magnetization curve at 5 K for single LSMO and ESMO reference layers and LSMO/ESMO bilayer.

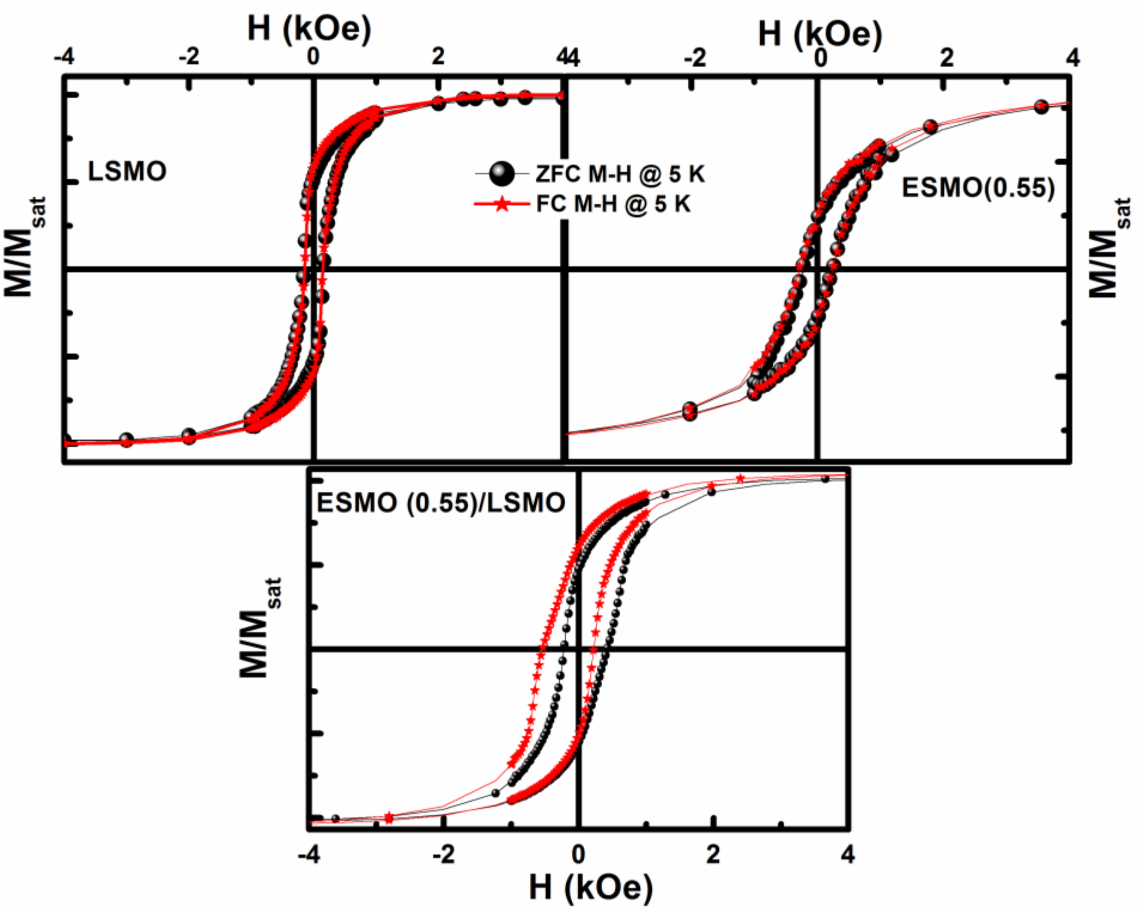

Figure S(4): Isothermal M-H loop data taken at 5 K after cooling the sample under ZFC and FC of 6 kOe for single LSMO and ESMO reference films and comparison with LSMO/ESMO bilayer.
